# A capital-based approach to better understand health inequalities: Theoretical and empirical explorations

**DOI:** 10.1016/j.ssmph.2022.101309

**Published:** 2022-12-05

**Authors:** Yuwei Qi, J. Cok Vrooman, Josué Almansa, Patricia Ots, Sandra Brouwer, Sijmen A. Reijneveld

**Affiliations:** a University Medical Center Groningen, University of Groningen, Department of Health Sciences, Groningen, the Netherlands; b Department of Sociology/ICS, Utrecht University, the Netherlands; c The Netherlands Institute for Social Research|SCP, the Netherlands

**Keywords:** Public health, Health inequalities, Capital, Resources

## Abstract

**Background:**

The persistence of health inequalities may be driven by differences in education and income, but also by other economic and non-economic factors. Our aim was to explore how the association between single-dimensional health and socioeconomic status (SES) changes when including health-related person capital, economic capital, social capital, cultural capital and attractiveness and personality capital.

**Methods:**

We used a capital-based approach to understand health inequalities. It presumes intertwined relationships between broadly measured health (‘health-related person capital’) and embodied resources (‘attractiveness and personality capital’) on the one hand, and ESC capital, i.e., economic, social, and cultural resources on the other. We used cross-sectional data on 152,592 participants from the Dutch Lifelines cohort study and estimated correlations using partial least squares structural equation modelling.

**Results:**

The correlation between SES and health-related person capital (r = 0.15) was stronger than the correlations between SES and single-dimensional health (physical and mental health; r = 0.12 and r = 0.04, respectively). ESC capital, combining economic, social and cultural capital, showed a correlation of 0.34 with health-related person capital. This was stronger than the correlation between health-related person capital and economic capital alone (r = 0.19). Lastly, the correlation between health-related person capital and ESC capital increased when health related, attractiveness and personality resources were combined into a single person capital construct (from r = 0.34 to r = 0.49).

**Conclusions:**

This exploratory study shows the empirical interconnectedness of various types of resources, and their potential role in the persistence of health inequalities. Our findings corroborate the idea of considering health as a multidimensional concept, and to extend conventional SES indicators to a broader measurement of economic and non-economic resources.

## Highlights

• Socioeconomic inequalities in health should be addressed as result from unequal distribution of various forms of resources.
• Myopic or simplistic health policies should not only focus on the unequal distribution of economic resources.

## 1 Introduction

Research consistently confirms that socioeconomic health inequalities have increased in recent years, and policy interventions aiming to tackle its persistency have not been able to reverse this trend (Mackenbach, 2019). Social-epidemiological explanations of health inequalities traditionally take the existence of social inequalities as a given. They ignore the fundamental questions of why social inequalities exists and why these are so persistent (Bourdieu, 1989). Moreover, these explanations are based on a rather static and unidirectional assessment of the relationship between socioeconomic status (SES) and health. Incorporating more sociological theory into the way that we conceptualize people's social position may enable us to gain a better understanding of how and why health inequalities develop(Bourdieu, 1986). This information can subsequently be used in social policy measures that aim to tackle these inequalities.

One of the well-known approaches to conceptualize social disparities was introduced by Pierre Bourdieu. He argued that the social hierarchy reflects the distribution of three forms of capital, (Bourdieu, 1986, 1989, 2013): economic, social, and cultural capital. In Bourdieu's perspective these three forms of capital are correlated and may account jointly for the production and reproduction of inequalities between groups of individuals (Abel, 2008; Abel & Frohlich, 2012; G. Veenstra & Abel, 2015, 2019) and thus sustain social class differences. In this study we do not aim to ascertain the segmentations of a capital-based social class structure (Savage, 2015; Savage et al., 2013), but to position and analyse health inequalities within various types of resources.

Out of these three types of resources, economic capital is captured in part by conventional measures of socioeconomic status (SES) (such as income, education and occupational status), but these may not provide full coverage of their position in economic terms (Galobardes et al., 2006). While income is a common measure of SES, it does not cover all the monetary resources people possess. Their wealth (i.e., liquid assets and the net value of the houses they own) could also be important for their health. These can be used to buffer the effects of income losses due to unemployment or illness, enable people to pay for non-insured medical treatment and prevention, and can reflect power or influence over others (Braveman et al., 2005). Conceptualizing economic capital by combining more extensive measures of monetary resources with traditional SES measures may therefore shed more light on the relationship the various socioeconomic resources people hold and health inequalities.

Other types of capital are also likely to contribute to the existence of health inequalities; and incorporating these in the theoretical and empirical framework could therefore affect the association between socioeconomic position and health (Bourdieu, 1986, 1989, 2013; Lin, 1999, 2017, pp. 3–28; Vrooman, 2016; Vrooman et al., 2014). These other types of capital in particular include non-material resources in the form of social and cultural capital (Bourdieu, 1986; Savage, 2015). Social capital refers to the investments, access and mobilization, and returns that relate to resources that are embedded in social ties (Bourdieu, 1989). Social capital is essential for constructing individual health via family members and other social ties. Cultural capital refers to resources founded in dominant symbolic and cultural systems (Bourdieu, 1986, 2013). It may play a significant role in understanding how culture shapes health inequalities and health-related help-seeking practices (Abel, 2008). More comprehensive measuring of health in itself can also affect the associations between SES and health. First, health can be regarded as a multidimensional concept and as such recognizes more than simply the absence of disease. It includes well-being across physical, mental, as well as social domains (Huber et al., 2011). On top, research shows disparities in the way different social classes conceptualize health (Tubeuf et al., 2008, p. 24) (Stronks et al., 2018). For example, in lower socioeconomic groups, individuals emphasised health as the absence of sickness, while higher socioeconomic groups tended to define health in terms of vitality (D'Houtaud & Field, 1984). It is therefore valuable to analyse more than one domain of health, in keeping with the WHO's broad definition of health ("Ottawa charter for health promotion," 1986).

**Fig. 1:**
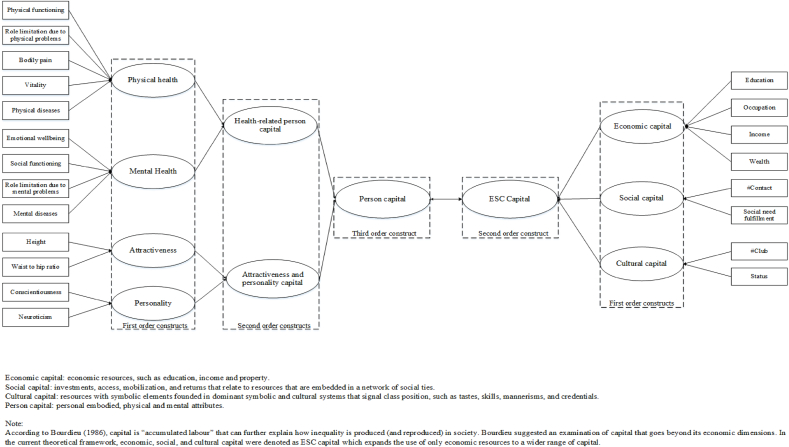
Theoretical model of the capital relations. Observed measurements in squares, and latent constructs in circles.

Moreover, health has been considered as an important form of capital that people can use and invest in (Grossman, 1972; Vrooman, 2016; Vrooman et al., 2014) Mackenbach (2019) states that health plays a key role in the allocation of individuals to social positions. In his view, social inequality does not only cause health disparities; the latter in turn may aggravate social distinctions, creating vicious cycles. The fact that health acts as a stock of biopsychosocial resources that people can draw on to participate in society also highlights health as an asset (Williamson & Carr, 2009). In this approach, socioeconomic health inequalities can be re-conceptualized as the association of different types of resources, i.e., person capital and economic capital. Person capital then comprises not only a wide notion of health but also other forms of ‘embodied’ individual differences that may be non-health-related. Traits like attractiveness and personality have been conceived as a relevant domain of non-material resources contributing to inequalities in health (Anyzova, Petra, & Mateju, 2018). For instance, personality has been linked to the development of chronic diseases, which in turn can influence personality development, via the experienced limits and constraints in functioning and activities (Brouwer et al., 2020; Sutin et al., 2013). As such, person capital refers to various sorts of embodied personal attributes, such as physical health, mental health and appearance. Our notion of person capital originated from a study conducted by the Netherlands Institute for Social Research(Vrooman et al., 2014). Person capital is seen as the 'fourth inequality dimension', next to economic, social and cultural capital. Fig. 1 displays the framework as based on Bourdieu's theoretical notions with the addition of this fourth dimension based on Vrooman et al. (2014). Studies that conceptualize social disparities using multiple forms of capital may lead to the identification of new routes to intervene upon health inequalities. However, research on this topic is very scarce, in particular regarding various personal attributes as important resources (Abel, 2008; G. Veenstra & Abel, 2015) (Pinxten & Lievens, 2014). Our theoretical framework considers the association of ‘person capital’ with ‘ESC capital’ (Fig. 1). This model assumes health to be part of person capital, jointly with other person-bound factors such as attractiveness. The total of person capital is related to ESC capital, i.e., people's economic, cultural and social resources. This ESC capital construct expands the use of only economic resources to a wider range of capital.

Using the proposed framework, the aim of this study was to explore how the association between single-dimensional health and SES changes when including more comprehensive measures of health (i.e., health-related person capital), economic capital, social capital, cultural capital and attractiveness and personality capital.

## 2 Methods

### 2.1 Cohort design and study population

The study was conducted using data from the Lifelines Cohort Study (Scholtens et al., 2015; Stolk et al., 2008), merged with registry data from Statistics Netherlands. Lifelines is a multi-disciplinary prospective population-based cohort study examining in a unique three-generation design the health and health related-behaviours of 167,729 persons living in the North of the Netherlands. It employs a broad range of investigative procedures in assessing the biomedical, socio-demographic, behavioural, physical and psychological factors which contribute to the health and disease of the general population, with a special focus on multi-morbidity and complex genetics. Recruitment and data collection have been described extensively elsewhere (Scholtens et al., 2015). In short, eligible participants, i.e. those aged 25–49, and their family members were recruited through invitations by their general practitioner based on the practice register; in the Netherlands all citizens have to be registered at a general practice. In addition, there was an option to self-register. Lifelines was conducted according to the guidelines in the Declaration of Helsinki and all procedures involving human subjects were approved by the Medical Ethics Committee of the University Medical Center Groningen. Written informed consent was obtained from all participants. Lifelines has been shown to be representative for the population of the north of the Netherlands which is faced with relatively pronounced problems, related to their low socioeconomic position compared to the entire country(Klijs et al., 2015) (van Zon, 2017). Within the Lifelines population, absolute and relative socioeconomic health inequalities differed across age groups by indicator of socioeconomic position, health outcome, and gender. Absolute inequalities were most pronounced for mental health by household income while relative inequalities were most pronounced for physical health by educational level (van Zon et al., 2015). The current study used a subsample of 152,592 participants aged 18 years and older, who visited the research centres between November 2006 and March 2013 for the baseline measurements. Data from the Lifelines cohort were further enriched by the linkage with Statistics Netherlands (Central Bureau voor de Statistiek; Dutch acronym: CBS). Each participant has an internal and secure key that facilitates linkage to the CBS datasets (Bakker et al., 2014).

### 2.2 Measures

**Person capital** was assessed as *health-related person capital and attractiveness and personality capital.*

*Health-related person capital* regarded physical health, mental health, and the number of diseases. Physical and mental health were measured using seven subscales of the Dutch version of the SF-36 Health Survey, together with the reported number of diseases. The SF-36 subscales included were: (i) physical functioning (10 items); (ii) role limitations due to physical problems (four items); (iii) bodily pain (two items); (iv) vitality (four items); (v) emotional wellbeing (five items); (vi) social functioning (two items); (vii)) role limitations due to emotional problems (three items). The SF-36 is a reliable and validated instrument, with scores on each of the subscales ranging from 0 to 100, where higher scores reflect better health status (Hays & Morales, 2001). Number of physical and mental diseases was measured using self-report questionnaire, which included eighteen physical diseases and six mental diseases based on a report from the Netherlands National Institute for Public Health and the Environment (see Appendix 1). (Gijsen et al., 2013).

*Attractiveness and personality capital* was measured by proxies of attractiveness and personality. Due to data availability, individuals’ height (in cm) and waist-hip ratio (WHR) were used as proxies for attractiveness. Height, waist circumference and hip circumference were measured using anthropometric measures at the Lifelines research centres. Height was standardized within gender (Tulin et al., 2018; Valentova et al., 2014). WHR was first calculated as waist measurement divided by hip measurement, and then categorized into six levels, ranging from 0 to 5, where a higher level indicates more attractiveness (Furnham et al., 1997). The categorization differs for male and female, as Furnham and Moutafi (2002; 1997) posited that WHR interacts with sex where attractiveness is concerned. For males, a WHR of 0.85–0.90 has been assigned with the highest score; for females this concerned a WHR of 0.70–0.75 (Furnham et al., 1997, 2006). Personality was measured through the Dutch Revised NEO Personality Inventory (NEO-PI-R); specifically we used items for neuroticism and conscientiousness (Hoekstra et al., 1996). These two personality traits were selected based on their influence on psychopathology (according to the NEO manual) and were defined and illustrated with sample items in Appendix 2 (Kotov et al., 2010; Lamers et al., 2012). All sixty-four items were answered on a five-point Likert-type scale that ranged from strongly disagree (1) to strongly agree (5). Each facet scale score reflects the sum of scores on the eight corresponding items, thus ranges from 8 to 40. Sum scores were calculated when ≥5 valid item scores were available, and adjusted for the number of missing items. The total score of each domain is the sum of all items from each subscale, with higher scores indicating stronger trait (Distel et al., 2009). The authorized Dutch translation of this scale has good reliability and validity (Ganzeboom & Treiman, 2003, pp. 159–193).

**Economic capital** included educational level, occupation, income, and wealth. *Educational level* was based on participants’ highest level of completed education and options were merged into (1) no education, (2) low (primary education; lower or preparatory secondary vocational education; junior general secondary education), (3) intermediate (secondary vocational education or work-based learning; senior general secondary education, pre-university secondary education), and (4) high (higher vocational education; university education). *Occupation* was assessed as occupational group membership, which was measured at baseline by asking participants about their occupation and the main tasks related to their occupation. CBS coded all occupations automatically according to the International Standard Classification of Occupations (ISCO) 08 (International Labour Organization, 2012). The resulting codes were then converted to the International Socio-Economic Index of Occupational Status (ISEI-08). The details for generating the occupational status measures are described in Ganzeboom and Treiman (Ganzeboom & Treiman, 2003, pp. 159–193). *Income* and *wealth* were measured through registry data from the CBS. Standardized disposable household income was computed in percentiles. Wealth was measured by the housing value and other types of wealth. Wealth other than housing value was calculated by total household asset minus the housing value. Standardized wealth measures were computed in percentiles.

**Social capital** was measured through number of social contacts and social need fulfilment. *Number of social contacts* was assessed by asking participants to report the number of different persons with whom they had contact on average within two weeks’ time (continuous scale). People were instructed only to count those contacts in which personal matters were exchanged or discussed, either through written or oral communication. *Social need fulfilment* was assessed using the six items on social well-being from the short version of the Social Production Function Instrument for the Level of well-being (SPF-IL). These six items of the SPF-IL assess affection (three items) and behavioural confirmation (three items) (see Appendix 3). A total score was computed with higher scores indicated better need fulfilment.

**Cultural capital** was measured by participation in organized clubs and groups and perceived status. *Participation in organized clubs and groups* was measured using a continuous variable (range 0–6) indicating the number of participations in sports clubs, neighbourhood or social clubs, political parties, patient associations, church or religious communities, and other clubs. *Perceived status* was assessed using the three items on status from the short version of the Social Production Function Instrument for the Level of well-being (SPF-IL) The scores range from 0 to 6, with higher scores indicating a better situation (see Appendix 3).

**Demographic information** regarded age, gender and marital status (having a partner, single, widow(er)/divorced and other). Information on these variables was self-reported.

### 2.3 Conceptual model of the capital-based approach

Our theoretical model is depicted in Fig. 1, in which circles represent the latent capital constructs in the structural measurement model, and rectangles depict their formative indicators. This model includes two parts. First, the measurement part specifies the relations between latent capital constructs and their observed indicators, which have been described in the “measures” section. Second, the structural part specifies the relationships between the capital constructs. The structural model assumes health to be part of person capital, jointly with other person-bound factors such as attractiveness and personality. Thus, health-related person capital is a second-order construct formed by the physical and mental health constructs; and the combination of attractiveness and personality also forms a second-order construct. The total of person capital is a third-order construct, combining health related, attractiveness and personality resources. On the other side, the second-order ESC capital encompasses the economic, cultural and social capital constructs. Finally, the constructs person capital and ESC capital are correlated.

### 2.4 Statistical analysis

First, we described all the variables with frequencies for categorical variables, and means and standard deviations for the continuous measures. Next, we assessed inter-correlations of different capital constructs using a partial least squares structural equation modelling (PLS-SEM) approach, which handles formative constructs, to address the associations as described in Fig. 1 (Tenenhaus et al., 2005). The latent capital constructs were estimated as weighted linear combinations of their indicator variables, and standardized (zero mean and standard deviation equal to 1). These weights represented the relative contribution of the indicator to the definition of its corresponding latent capital construct. For the structural part, we estimated correlations between the capital constructs. Given that capital constructs are standardized, the regression coefficient between constructs equals to their correlation – without implying directionality in its effects.

The second order constructs (i.e. health-related person capital, attractiveness and personality capital, and ESC capital as depicted in Fig. 1), were obtained as follows: first we estimated a PLS-SEM model with all the first order constructs correlated among themselves, then we calculated the construct scores with fixing of the weights from the measurement part, and finally we used these new scores as indicators of the second order construct. Additional orders followed the same strategy (i.e., using second order construct as indicator variables of the third order). Given that weights were fixed, lower order construct scores (and correlations among them) remain invariant when creating the higher-order constructs into the same model. We estimated the PLS-SEM models based on pairwise correlations, so individuals with missing data contributed with their non-missing variables. To test the significance of the path coefficients and the loadings, create confidence intervals were estimated with bootstrapping.

The analytical strategy consisted of estimating PLS-SEM models consecutively by adding building blocks. Four models were constructed for the first building block: Model 1a included physical health, mental health, and SES (education, occupation and income), all three constructs correlated among them. In Model 1b, we added a second-order construct based on physical health and mental health, to create health-related person capital, correlated with SES. Model 1c extends Model 1a by adding wealth to SES in the construct of economic capital. Finally, Model 1d adds the second order health-related person capital construct, as in Model 1b correlated with economic capital. The second building block regarded adding social and cultural capital to Model 1 by creating an ESC capital construct from economic, social and cultural capital, leading to Model 2. The third building block regarded expanding person capital by including attractiveness and personality capital, as in Model 3, which corresponds to the full conceptual model depicted in Fig. 1. Before estimating the constructs, all capital indicators were rescaled to the same direction, meaning that higher scores always indicated a better state.

Finally, we explored differences in the constructs scores by comparing their means across age, gender, and marital status.

Descriptive statistics were performed in SPSS version 25, and PLS-SEM analyses were performed using R version 3.6.2 with the semPLS package (Monecke & Leisch, 2012).

## 3 Results

### 3.1 Descriptives of the sample

The majority of the study sample was female (58.5%) and had a partner (85.2%). Of the participating individuals, less than 10% had no education completed, 29.2% had a low educational level, 38.5% an intermediate level, and 29.1% a high educational level (see Table 1).

**Table 1:** Characteristics of the study sample.

	Mean (SD) or n (%)*	Valid cases
**Demographics**		
Age		
18-24	9021(5.9%)	152,592
25-34	25,829(16.9%)	
35-44	41,663(27.3%)	
45-54	43,521(28.5%)	
55-64	19,876(13.0%)	
65+	12,682(8.4%)	
Gender		152,592
Male	63,322(41.5%)	
Female	89,270(58.5%)	
Marital status		151,944
Have a partner	129,528(85.2%)	
Single	16,412(10.8%)	
Widow(er)/Divorced and other	6004(3.9%)	
**Construct and corresponding indicators**		
Physical health		
Physical functioning^a^	95.0(14.4)	148,383
Role limitation due to physical problems^a^	86.5(29.5)	148,389
Bodily pain^a^	84.4(19.2)	148,162
Vitality^a^	67.7(17.1)	148,489
Physical diseases	0.9(1.1)	152,592
Mental health		
Emotional wellbeing^a^	79.6(13.8)	148,487
Social functioning^a^	87.4(18.2)	148,509
Role limitation due to mental problems^a^	90.6(25.5)	148,389
Mental diseases	0.2(0.6)	152,292
Attractiveness		
Height^b^	174.8(9.4)	152,292
Waist to hip ratio score	2.1(1.3)	152,292
Personality		
Neuroticism^c^	47.1(12.8)	129.970
Conscientiousness^c^	63.1(9.8)	131,319
Economic capital		
Education		152,292
No education	860(0.6%)	
Low	44,582(29.2%)	
Intermediate	58,770(38.5%)	
High	44,461(29.1%)	
Occupation	46.1(21.0)	145,196
Income	54.6(26.8)	145,727
Housing wealth	50.0(28.9)	152,135
Other wealth	51.6(28.0)	152,314
Social capital		
Number of social contacts	20(29.7)	143,361
Social needs fulfilment^d^	12.6(2.7)	142,905
Cultural capital		
Number of clubs joined	1.3(0.8)	120,753
Perceived status^d^	3.3(1.6)	142,596

a SF-36 subscales.

b In the analysis height was gender standardized.

c Revised NEO Personality Inventory (NEO-PI-R).

d Social Production Function Instrument for the Level of well-being (SPF-IL).

### 3.2 Health-related person capital, SES, and economic capital

In the first building block we started by comparing correlations between single-dimensional health, SES and health-related person capital (Models 1a and 1b in Table 2). The correlation between the second order health-related person capital and SES (r = 0.15) was higher than any of the correlations between its first-order level constructs (physical and mental health), and SES (r = 0.12 and r = 0.04, respectively). This implied that the elaborated health measure resulted in a stronger correlation with SES. In Models 1b and 1d, the same pattern can be observed for health-related person capital and economic capital after adding wealth (r = 0.19 vs r = 0.15).

**Table 2:** Correlations and bootstrap 95% confidence intervals for health-related person capital, SES, and economic capital, Models 1a-1d.

	Mental health	SES	Economic capital
Physical health	0.74 ^a,c^	0.12 ^a^	0.13 ^c^
	[0.73, 0.74]	[0.11, 0.13]	[0.13, 0.14]
Mental health		0.04 ^a^	0.07 ^c^
		[0.03, 0.05]	[0.06, 0.08]
Health-related person capital		0.15 ^b^	0.19 ^d^
		[0.15, 0.16]	[0.19, 0.20]

^∗^a,b,c,d refers to models 1a-1d.

### 3.3 Health-related person capital, economic, social and cultural capital

In building block 2 we added social and cultural capital in the ESC capital construct. Within health-related person capital, physical health was very low correlated to social capital, and cultural capital (r = 0.06) (Table 3), and mental health was positively related to social capital (r = 0.28) but not to cultural capital. Within the ESC capital, economic capital demonstrated no correlation with social capital while it was positively correlated to cultural capital. Social capital also showed a positive correlation with cultural capital (r = 0.27). Health-related person capital itself showed the strongest correlation with social capital, next to economic capital. Overall, health-related person capital, as a multidimensional construct showed stronger correlation with economic capital and social capital, compared to its lower level constructs (physical and mental health). ESC capital, which included economic, social and cultural capital, showed a correlation of 0.34 with health-related person capital. This correlation was higher than the correlation between health-related person capital and economic capital alone (r = 0.34 vs r = 0.19).

**Table 3:** Correlations and bootstrap 95% confidence intervals for health-related person capital, economic capital, social capital, and cultural capital, Model 2.

	Mental health	Economic capital	Social capital	Cultural capital	ESC capital
Physical health	0.73	0.12	0.06	0.06	
	[0.72, 0.73]	[0.11, 0.13]	[0.05, 0.07]	[0.05, 0.07]	
Mental health		0.06	0.28	-0.02	
		[0.05, 0.07]	[0.27, 0.29]	[-0.03, -0.02]	
Economic capital			-0.01	0.26	
			[-0.02, 0.00]	[0.25, 0.27]	
Social capital				0.27	
				[0.27, 0.28]	
Health-related person capital		0.17	0.32	0.03	0.34
		[0.16, 0.17]	[0.32, 0.33]	[0.02, 0.04]	[0.34,0.35]

### 3.4 Person capital and ESC capital

Adding attractiveness and personality capital to the model of building block 3, we found that attractiveness and personality capital was associated with economic, social, and cultural capital (r = 0.25, r = 0.25, and r = 0.28, respectively) (Table 4). In the final phase of constructing person capital and ESC capital (including economic, social and cultural capital), we found that person capital was positively related to ESC capital, r = 0.49, 95%CI [0.49, 0.50]. Again, the correlation between person capital and ESC capital was stronger than the correlation between health-related person capital alone and ESC capital (r = 0.49 vs r = 0.34).

**Table 4:** Correlations and bootstrap 95% confidence intervals for third order person capital, economic capital, social capital, and cultural capital, Model 3.

	Attractiveness and personality capital	Economic capital	Social capital	Cultural capital	ESC capital
Health-related person capital	0.50	0.03	0.19	-0.01	
	[0.49, 0.50]	[0.02, 0.04]	[0.19, 0.20]	[-0.02, 0]	
Attractiveness and personality capital		0.25	0.25	0.28	
		[0.24, 0.26]	[0.24, 0.26]	[0.27, 0.28]	
Economic capital			-0.11	0.21	
			[-0.12, -0.10]	[0.20, 0.21]	
Social capital				0.21	
				[0.20, 0.21]	
Person capital					0.49
					[0.49, 0.50]

### 3.5 Measurement model for person capital constructs and ESC capital constructs

Both physical health and mental health contributed to the health-related person capital with weights 0.29 and 0.77 respectively (Table 5). For attractiveness and personality capital, the loading of attractiveness was low, although the bootstrap confidence interval was significant (given the relatively large sample size), it was not clinically relevant. Personality was the predominant factor for attractiveness and personality capital with a weight of 0.99. Social need fulfilment seemed to be predominant in constructing social capital. Lastly, perceived status seems to be the most influential indicator for cultural capital.

**Table 5:** Measurement model for person capital constructs and ESC capital constructs. Indicator Weights and bootstrap 95% confidence intervals.

	Health-related person capital	Attractiveness and personality capital	Economic capital	Social capital	Cultural capital
Mental health	0.77				
	[0.75, 0.79]				
Physical health	0.29				
	[0.27, 0.31]				
Attractiveness		0.09			
		[0.08, 0.10]			
Personality		0.99			
		[0.99, 0.99]			
Education			0.26		
			[0.24, 0.29]		
Income			0.74		
			[0.72, 0.76]		
Occupation			0.21		
			[0.18, 0.23]		
Wealth; house			0.09		
			[0.07, 0.12]		
Wealth; other			0.11		
			[0.09, 0.13]		
# Social contacts				0.15	
				[0.14, 0.17]	
Social need fulfilment				0.98	
				[0.97, 0.98]	
# Clubs					0.15
					[0.14, 0.17]
Status					0.98
					[0.97, 0.98]

Regarding the higher-order constructs, economic capital, social capital, and cultural capital all contributed to ESC capital with the highest weight of 0.72 for social capital (not shown in table). In the overall person capital construct, we observed that the combination of personality and attractiveness, with a coefficient of 0.83, appeared to be more influential than health-related person capital (0.28).

### 3.6 Differences in capital scores across demographic variables

Differences in possession of the various forms of capital were tested in three demographic groups (see Appendix 4). Regarding the health elements of person capital, men scored higher on both physical health and mental health. Individuals without a partner tended to score lower on all capital constructs. Finally, people aged 65 and over scored lower on economic and on cultural capital.

## 4 Discussion

The present study aimed to investigate how the association between single-dimensional health and SES changes when including more comprehensive measures of health (i.e., health-related person capital) and other types of capital. Our results confirmed that the proposed capital-based model, including health-related person capital, economic capital, social capital, cultural capital and attractiveness and personality capital, was able to capture socioeconomic health inequalities beyond the traditional link between SES and health. In particular, we found that treating health as a multidimensional concept and including other economic and non-economic resources strengthened associations compared to the association between unidimensional health and SES.

First, we found that multi-dimensional measurement of health (i.e. health-related person capital) led to a stronger relation between SES and health than measuring health with a one-dimensional measure. Our results suggest that health should be regarded as a multidimensional concept and as such recognizes that health is more than the absence of disease. This is in line with the idea of a positive health concept (Huber et al., 2011). An innovative aspect of our approach is that we regard physical and mental health as personal resources that may evoke societal advantages and disadvantages. Health is embodied in individuals, and, as such, it is not a directly tradable resource like economic capital (Williamson & Carr, 2009). On the one hand, health plays a role in allocating individuals to different access to various types of resources; having better health subsequently provides the basis for investing in and accumulating stocks of other types of resources(Mackenbach, 2019). For example, engagement in education and labour market provides people with opportunities to build up financial and social resources. On the other hand, ill health will limit one's participation in society, which might impede the acquisition and accumulation of other resources (Marcotte & Wilcox-Gök, 2001). It is therefore important to bring health, as a type of resource with multidimensional aspects, into the equation in understanding the mutual influence of health inequalities and other interconnected resources.

Second, the current study showed that a more elaborate measure of economic resources, including wealth, led to stronger associations between economic resources and health than using traditional SES measures. Moreover, we found that economic capital, together with social and cultural capital resulted in a stronger association between health-related person capital and ESC capital than the association between health-related person capital and only economic capital. These findings indicate that capital is not only of an economic nature, but also includes other resources that are socially valued (Abel et al., 2011). This is in line with an emerging literature. The importance of a wider notion of economic capital has been stressed before (Abel, 2008; Gerry Veenstra, 2002; Giordano & Lindstrom, 2010; Healy & Côté, 2001; Vincens et al., 2018). Social capital has been identified as an important predictor of physical and mental health and mortality(Ehsan et al., 2019). Increased support and fulfilling social needs may be important mechanisms through which health is promoted (Gönç Şavran, 2018; Kawachi & Berkman, 2000; Song, 2013). In addition, cultural capital has also received more attention in recent years as a predictor of both socioeconomic inequalities in general and health disparities (Abel, 2008; Büchner et al., 2012; Eikemo & Øversveen, 2019; Mackenbach, 2012, 2019). Bourdieu (1986) argued that cultural capital plays a key role in the emergence and passing on social differences. This relates to shared tastes and preferences, and attitudes that further linked to social class membership, reputation or fame. We found perceived status to be the most important indicator of cultural capital and club membership was less relevant. Although club membership inherently encompasses social contact, we have decided to add this factor under the cultural rather than social capital. In doing so, we follow the concept of cultural consonance. This refers to *the definition of* *Dressler (2017*, 2020)*: … degree to which individuals incorporate shared cultural knowledge into their own beliefs and behaviours*. *In the current study, the number of clubs people have joined indicates the extent to which they participate in behaviour that is valued in the dominant culture*. Cultural capital may act as a bearer of symbolic meaning, thus embodying an essential element of social hierarchy (Abel, 2008) that is reflected in the ESC capital construct.

Finally, we observed that an extension of the person capital with attractiveness and personality capital, i.e. attractiveness and personality, led to a further strengthening of the association between resources and health. In modern societies, social class is associated with individual personal characteristics, which may affect health (Mackenbach, 2010). The addition of attractiveness and personality capital adds insight into the role of psychological attributes and personal traits in explaining socioeconomic differences in health (de Graaf et al., 2012). Just like personality, attractiveness in general is also context-dependent: they are more valuable when they match the norms in the environment (Fietzer et al., 2016; Monocello, 2020; Naigaga et al., 2018). For instance, in cultures where women have limited economic opportunities, women with high levels of body fat were considered attractive, whereas the reverse is true for cultures that have an abundance of resources (Fisher & Voracek, 2006). This suggests that a certain type of appearance can be an advantage in some settings, but a disadvantage in others. It also means that 'more' is not necessarily 'better': a bodybuilder might have very developed muscles, but it could be contributed to the use of anabolic steroids and a fixation on training work, which does not always indicate a healthy state of body and spirit. It is therefore rather a question of the degree of alignment of personal characteristics with the demands of the environment. On the one hand, someone's position in society tends to be better if (s)he is in a good physical and mental state, attractive, and has a becoming personality (Furnham et al., 2006; Palmer & Peterson, 2021). On the other hand, being (un)successful also depends on other resources, relating to economic capital, social relations and cultural resources. The addressing of person capital highlighted the significance of factors other than conventional physical health measurements.

### 4.1 Strengths and limitations

The present study has some important strengths. It contributes to the discussion on social determinants of health by showing the need to include different forms of capital simultaneously in one model. The use of a large nationally representative sample with data collected on all different capital indicators enabled us to empirically explore the proposed framework. Moreover, PLS-SEM, as a second-generation technique, allowed the simultaneous modelling of relationships among multiple indicators and constructs. Therefore, a differentiation between dependent and independent variables is superfluous, in line with our assumption that capital constructs are inherently interlinked. The higher order capital constructs acted as a vehicle to re-arrange the indicators and/or constructs across different concrete sub-dimensions of the more abstract capital constructs.

Next, our study also has several limitations. First, due to cross-sectional nature of our data, we could not assess how people convert one form of capital into another over time. Longitudinal studies would offer interesting possibilities in this regard. Second, the measurement of certain resources was limited, especially with respect to attractiveness and personality capital, social and cultural capital. Regarding attractiveness and personality capital, we were only able to include two proxies for attractiveness and two facets of personality that may overlap with health-related person capital to some extent. The various indicators of person capital showed a fairly high correlation, which suggests that the health measures partially control for the health-related variation in attractiveness and personality capital. The proxies of attractiveness assumed attractiveness is based on body size, which partially relates to the health risks associated with being overweight and obese. Moreover, the attractiveness proxies could not take into account of variation in body ideals in e.g., sexual and gender minority groups*.* Third, some indicators were entirely based on self-reports (e.g. those relating to social and cultural capital), which may have invoked response bias. Finally, our study sample has an overrepresentation of middle-aged people due to the sampling strategy, and most Lifelines participants have been born in the Netherlands (97%) and have a Caucasian ethnicity (98%). The generalizability to other populations thus deserves further scrutiny.

### 4.2 Implications

The present study highlights the importance of considering various forms of capital to uncover class-related mechanisms that contribute to socioeconomic inequalities in health. Traditionally people have been thought of achieving good health by reaching a high income or occupational status. However, what seems to be equally important is the possession of other types of monetary and non-monetary resources. Public health authorities should therefore encourage individuals to develop the possession of more forms of capital, and to avoid their depletion. For example, aesthetic and mental aspects of person capital provide new opportunities for the development of policies to promote labour market opportunities. Public provisions, such as health care or tax deductions for medical costs, are theoretically important for the development and maintenance of person capital (availability of medicines and treatments for physical and psychological complaints, orthodontics, cosmetic treatment). In part, aesthetic and mental characteristics as fixed as these attributes are difficult to adjust and may to some extent be genetically determined. One's mental and aesthetic characteristics, however, are not entirely unchangeable; e.g., clothing choice and grooming. This provides starting points for effective interventions, as a supplement to the existing health policy. Generally speaking, policy measures should at least tackle the capital disparities that are affecting the life chances of people with the least resources, and may thus also have relevance for the discussion on social calls differences.

In addition to strengthening people's economic capital, the size and quality of people's social networks and their engagement in cultural activities and other forms of cultural capital should be a target for future interventions. Regarding future research, our findings should encourage researchers to include more intricate measures of attractiveness and personality capital, social capital, and cultural capital to further develop the capital-based approach pertaining to health inequalities. For example, the argument about personality should be strengthened by using other personality measurements that are less associated with health outcomes. Next, to account for the intersectionality of disparities by gender, race, age, etc., we recommend future research to explore if there are differences in the interrelations between the four forms of capital across various socio-demographic groups (Axelsson Fisk et al., 2018; Evans et al., 2018; McCall, 2005) Finally, the relationships between health resource disparities and other forms of capital may vary between countries, depending on their cultural and structural background (e.g. diverging health preferences, different norms and conventions among health practitioners, national welfare regimes). This stresses the need for further comparative approaches.

To conclude, our capital-based approach suggested that socioeconomic inequalities in health may not be merely driven by education and income, but also by other economic and non-economic factors. Our findings corroborate that it is worthwhile to consider health as a multidimensional concept, and to extend conventional SES indicators to broader measures of economic and non-economic resources. This may offer a route to further disentangle socioeconomic differences in health, and to contain or reduce these in a more effective manner.

## Ethical statement

Lifelines was conducted according to the guidelines in the Declaration of Helsinki and all procedures involving human subjects were approved by the Medical Ethics Committee of the University Medical Center Groningen. Written informed consent was obtained from all participants.

## Funding

This research did not receive any specific grant from funding agencies in the public, commercial, or not-for-profit sectors.

## Author contributions

YQ, CV, JA, PO, SB, and SAR conceptualized and designed this study, had full access to and verified the underlying data, drafted the initial manuscript, and reviewed and revised the manuscript. YQ and JA carried out the analyses and reviewed and revised the manuscript. YQ, JA, CV, PO, SB, and SAR reviewed and revised the manuscript. All authors read and approved the final manuscript as submitted and agree to be accountable for all aspects of the work.

## Declaration of competing interest

None.

## Figures and Tables

**Table:** 

**Physical diseases**	Arrhythmia
	Asthma
	Cancer
	Chronic obstructive pulmonary disease
	Coronary heart disease
	Dementia
	Diabetes mellitus
	Eczema
	Epilepsy
	Hearing disorders
	Heart failure
	Migraine
	Neck and back hernia
	Osteoarthritis
	Osteoporosis
	Parkinson's disease
	Rheumatoid arthritis
	Stroke

**Mental diseases**	ADHD
	Anxiety disorders
	Depression
	Overstrain and burnout
	Schizophrenia
	Other mental limitations

**Table:** 

Personality Domain and Facet Traits*	Definition of High Score
Neuroticism	Prone to negative emotions and pessimism
*Hostility*	Angry, irritable, frustrated, and bitter
*Self-consciousness*	Prone to shame, feeling uncomfortable and inferior in company
*Impulsiveness*	Inability to control cravings and urges
*Vulnerability to stress*	Dependent, hopeless and panicky when stressed

Conscientiousness	Organized, planful, and self-disciplined
*Competence*	Capable, sensible, prudent, and effective
*Self-discipline*	Task focus, perseverance, and self-control
*Deliberation*	Cautious, deliberate, and considerate

* The original complete NEO-PI questionnaire contains 240 items: eight items per facet, six facets per domain. An abbreviated 64-item version of the NEO was used in the Lifelines study, containing the two domains and seven facets. Data on the facets included in this table were the only available personality traits in the Lifelines Cohort Study. Descriptions derived from Costa and McCrae (2006).

**Table:** 

Capital indicator	Measurement	Question
Social capital: Social need fulfilment		
	Affection	Do people pay attention to you?
		Do people help you if you have a problem?
		Do you feel that people really love you?
	Behavioural confirmation	There are situations in which we deal with groups of people, for example at home, at work or during our leisure time. Do others appreciate your role in the group?
		When you are at school, at work, with family, at an association or in church, do you feel like you belong?
		Do others appreciate the things you do?
Cultural capital: Status		
	Status	Do people think you do better than others?
		Do people find you an influential person?
		Are you known for the things you have accomplished?

**Table:** 

	Health- related person capital	Attractiveness and personality capital	Economic capital	Social capital	Cultural capital
**Gender**					
Male	0.19	0.20	0.38	−0.05	0.28
Female	−0.13	−0.14	−0.29	0.04	−0.20
**Age**					
18-24	−0.04	−0.30	−1.01	0.17	−0.04
25-34	−0.07	−0.09	0.03	0.11	−0.02
35-44	−0.04	0.02	0.10	−0.01	−0.00
45-54	−0.02	0.07	0.15	−0.06	0.04
55-64	0.10	0.04	0.09	−0.05	0
65+	0.23	−0.03	−0.35	−0.01	−0.04
**Marital status**					
Having a partner	0.05	0.03	0.03	0.02	0.01
Single	−0.28	−0.22	−0.21	−0.12	−0.03
Widow(er)/Divorced and other	−0.42	−0.14	−0.11	−0.06	−0.09

* The capital scores are constituted by the value of the standardized variable value and the weight of the corresponding variable in Model 3. The estimation is non-parametric.

## Data Availability

The authors do not have permission to share data.
